# Exploring the self-preparedness of frontline healthcare workers in a low- and middle-income country from a humanitarian context during the COVID-19 pandemic: A constructivist grounded theory study

**DOI:** 10.3389/fpubh.2023.1043050

**Published:** 2023-03-27

**Authors:** Farzana Khan, Tasnim B. Azad, Saiduzzaman Bhuyian, Hasina Karim, Liz Grant

**Affiliations:** ^1^Global Health Academy, University of Edinburgh, Edinburgh, United Kingdom; ^2^Fasiuddin Khan Research Foundation, Dhaka, Bangladesh

**Keywords:** humanitarian settings, frontline health care workers, Rohingya refugees in Cox's Bazar, self-preparedness, COVID-19 pandemic, low and middle-income countries (LMICs), forced displacement, palliative care

## Abstract

**Background:**

While research has been conducted on the availability, accessibility, and affordability of personal protective equipment for healthcare workers during the COVID-19 pandemic, little information is available on the ways in which health workers, especially those in humanitarian settings see themselves, and engage in self-preparedness for social, physical, and mental health and practical care in the pandemic. We sought to address this gap.

**Methods:**

We followed a constructivist grounded theory approach to guide in-depth interviews with 30 frontline doctors, nurses, and community healthcare workers recruited from the Rohingya refugee camps in Bangladesh using the purposive and snowball sampling methods. Analyses were carried out through the identification of codes in three phases: an initial line-by-line open coding, then focused axial coding, and finally selective coding.

**Findings:**

An emergent-grounded theory of “Navigating Self-Preparedness through Pandemics” was developed as we built a five-phased theoretical framework examining health worker responses with the following pillars: (a) pandemic shock; (b) pandemic awareness; (c) pandemic learning; (d) pandemic resilience, and (e) pandemic resurgence.

**Interpretation:**

The theory emerged as a realistic, socially, and culturally sensitive COVID-19 strategy to support healthcare workers. Self-preparedness was characterized by two interwoven processes: (1) the experiences of the daily life span of healthcare workers attempting to improve their own protection using all their potential while providing care for patients in a vulnerable setting and time and (2) the inseparable role of physical, psychological, social, and spiritual factors in each stage of learning during the pandemic to achieve better outcomes.

## Introduction

Self-preparedness is a term used to describe a set of key skills and competencies that are essential for healthcare professionals working in inter-professional teams (1). The capacity to manage oneself and others, deal with ambiguity and uncertainty, communicate effectively, think critically, participate in ongoing learning, recognize and evaluate problems, and exhibit empathy are among these abilities. Quality care protects health worker lives and the lives of patients, but quality care is not a given. It requires planning, and resources, and investment not just in the skills and knowledge of health workers but in their emotional, physical and social wellbeing. Evidence shows that an inter-professional team with multiple competencies is key to deliver high quality care (2). Self-preparedness is closely linked to the quality of care provided by healthcare professionals. When healthcare professionals possess the competencies of self-preparedness, they are better equipped to provide high-quality care to their patients.

Healthcare professionals supported to deliver care are able to provide high- quality care, and to share good practice, those not supported often feel isolated and at risk of burn out (3). Health workers in humanitarian settings were already struggling to deliver quality care, before the pandemic. The Lancet Global Health Commission on High-Quality Health Systems revealed that suboptimal quality of care contributes to 60% of deaths stemming from health conditions that could be addressed through healthcare (4). As per the World Health Organization's estimation, the COVID-19 pandemic has led to the loss of at least 180,000 healthcare workers' lives between January 2020 and May 2021 (5). Many reasons contribute to this high death toll including insufficient resources, lack of treatment, infection, burn out and stress. The lack of investment in the health workers who were sustaining systems under immense threat was particularly acute. Areas that were already under pressure became even more exposed.

Such an area is palliative care. The 2020 global Atlas of Palliative Care noted that a significant barrier for the provision of high-quality care is limited teamwork and insufficient skills and capacities of health workers (6). Evidence-based benefits of inter-professional teamwork include improved care quality, patient safety, and job satisfaction. Inter-professional teamwork involves different health and/or social professions who share a team identity and work closely together in an integrated and interdependent manner to solve problems and deliver services. For recognizing and correcting problems that could impair patient care, the capacity to recognize and understand difficulties is essential. In order to provide more patient-centered care, healthcare personnel might benefit from practicing empathy by better understanding the needs and concerns of their patients. Little is known about how humanitarian settings are supported, despite these being areas, where an estimated 2 billion people live and by 2030 nearly 50% of world's poor people will be living there (7). The COVID-19 pandemic has had overwhelming impact on self-preparedness of frontline healthcare workers (FHCWs) operating in a humanitarian environment.

To date, no research study has explored the processes of the self-preparedness of frontline healthcare workers (FHCWs) to deliver high-quality care in the humanitarian context, especially during pandemics. While much has been written and researched on the availability, accessibility, and affordability of personal protective equipment (PPE) amid the pandemic, there is very little evidence on how FHCWs see themselves and engage in self-preparedness on different levels, including, social, physical, mental, spiritual, and practical factors. We carried out this research to better understand the existing knowledge on self-preparedness among FHCWs working in a refugee camp, their understandings, particular needs, and how they valued themselves during the early period of the pandemic with probable human-to-human transmission. This research was an opportunity to contribute to the COVID-19 Research Roadmap of WHO to provide essential information on FHCWs' perceived knowledge and behavior regarding self-preparedness during a pandemic.

We sought to develop a substantial theoretical framework that explains the processes and factors for engaging in self-preparedness. Our fundamental question was “What are the processes of self-preparedness of FHCWs for quality care provision during the pandemic in Rohingya refugee camps?”

## Methodology

### Study design

This qualitative study employed a constructivist grounded theory approach, which emphasizes the role of the researcher in constructing knowledge through interaction with the research participants. The study is rooted in an interpretivist paradigm, suggesting that individuals create their own meaning and understanding of the world around them. The ontology of the study is relativist, acknowledging that reality is seen as subjective and context-dependent, while its epistemology is subjectivist, recognizing that knowledge is constructed through individual's experiences and perceptions. The study further draws on the theoretical framework of symbolic interactionism, which highlights the importance of social interaction and communication in shaping individuals' behavior and actions. The study's goal was to uncover the processes captured by interviews with FHCWS, as they continually modifying their behavior and actions within their social milieu and in the process reconstructing reality through daily management of their condition.

### Settings and participants

A total of 30 practicing frontline doctors, nurses, and community healthcare workers (CHWs) employed in the primary health centers of Rohingya camps, Cox's Bazar were interviewed. We excluded FHCWs not directly providing patient care such as managers or trainers and potential candidates who were unwilling to be involved in the study. Participants were selected initially using convenience sampling (8). Later, snowball sampling was used to identify potential participants (9).

Ethical approval was obtained from the University of Edinburgh and the Bangladesh Child Health Research Institute, and permission to carry out the research in the Rohingya camps was granted by the Civil Surgeon's Office, Cox's Bazar (CXB), and the Refugee Repatriation & Rehabilitation Commissioners' (RRRC) Office, CXB.

Informed consent was obtained verbally given the resource challenges of the camps and the concerns for identification. The initial general information which outlined the project was shared with the community, and a specific participant information sheet was given to those who met the eligibility criteria and expressed interest to participate in the study following that. Anonymity and confidentiality were taken into consideration throughout this study. When the participants volunteered and consented, an interview time which was convenient for their needs and the context was set. After the interview, the research assistants anonymized the audio-recorded interviews using code numbers and transcribed them.

Anonymized feedback was given to all members of the research setting (which included local hospitals, primary healthcare facilities, and non-governmental organizations). Participation was on a voluntary basis and participants could withdraw from the study at any point of the study, decline to answer any questions, or refuse to be audio-recorded during the interview.

### Data collection and analysis

Data were collected through individual online semi-structured interviews between May 1 and June 30, 2020. Because of travel restrictions, interviews were conducted online rather than face-to-face. This means the responses are reflective of the early phases of the COVID-19 pandemic. Interviews were digitally audio-recorded, transcribed verbatim, and checked for accuracy by FK. The first author, FK, mainly analyzed the data with independent contributions from all authors.

Data analysis was followed by iterative strategies in grounded theory with a focus on the processes, paying specific attention to time and context with simultaneous collection and analysis of data, manual coding, comparative analysis (within cases and across cases), theoretical sampling to refine theoretical ideas, memo writing, and the integration of theoretical frameworks into developing the grounded theory (10, 11). Coding involved (1) an initial phase (open coding) line-by-line coding, (2) a focused (axial coding), and (3) selective coding (theoretical coding) (12). We examined each transcript line by line to gain a closer look at what participants have said and to identify implicit concerns and explicit statements (13). Each interviewer wrote memos immediately after the completion of the interviews.

## Results

Table 1 shows the characteristics of the study sample. The following is a summary of the key characteristics of the participants in this study: A total of 30 participants, consisting of medical officers, nurses, nurse leads, and community health workers, were included in this study. The participants' years of experience ranged from 2 months to 3 years, with a mean of 1.4 years. The majority of participants were male (63.3%) and their ages ranged from 19 to 34 years old, with a mean of 26.1 years. The most common job roles among the participants were medical officers (33.3%) and nurses (30%), followed by community health workers (23.3%). Overall, the participants in this study represent a diverse group of healthcare workers with varying levels of experience and job roles.

**Table 1:** Characteristics of the sample of 30 frontline healthcare workers.

**Total participants (*****N*** = **30)**	
**Sex**	
Male	18 (60%)
Female	12 (40%)
Age, years^*^	26(19-34)
**Profession**	
Doctors	10 (33.3%)
Nurses	10 (33.3%)
Community health workers	10 (33.3%)
Years of experience in humanitarian camps^*^	16 months (1.5-36 months)

Data are n (%) and median (range).

* At time of interview participation.

**Figure 1:**
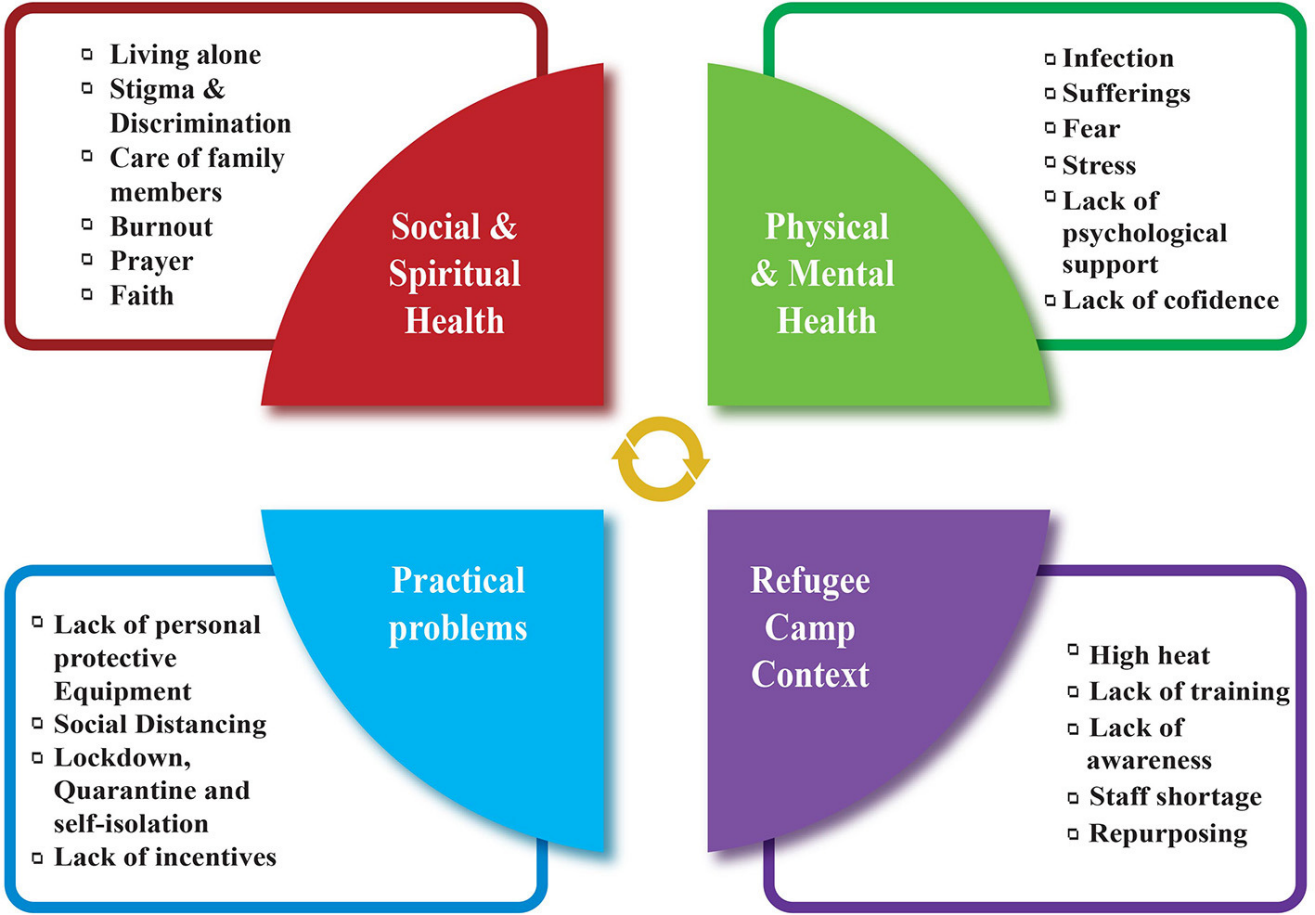
Multidimensional factors related to COVID-19 that affects FHCWs.

A set of consequences of the impact of COVID-19 on frontline health teams included managing self and others, coping with ambiguity and uncertainty, communication, critical reasoning, continuing own education, identifying and analyzing problems, and practicing empathy (Figure 1). Linking under the construct “self-preparedness,” we identified that these consequences led to a sense of self that is fundamentally different from that before the pandemic.

**Figure 2:**
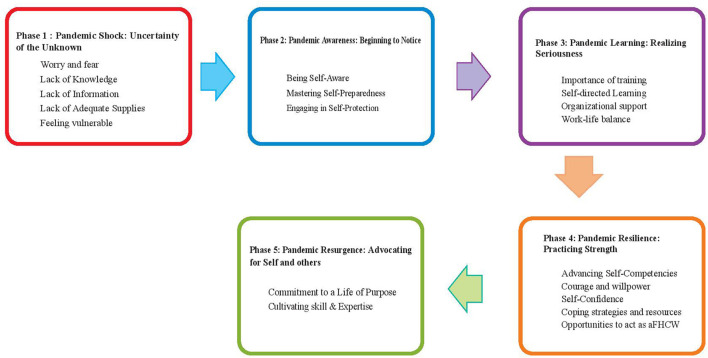
Five phases of the theoretical frame.

Examining how healthcare workers constructed their sense of self-preparedness provided the basis for a theory of care. Anonymized feedback was given to all members of the research settings, which included local hospitals. Healthcare workers working in this humanitarian setting moved from being confronted by fearful challenges to self-confidence during the pandemic. Data showed five phases (Figure 2) with the following categories and set of concepts: (a) pandemic shock; (b) pandemic awareness; (c) pandemic learning; (d) pandemic resilience, and (e) pandemic resurgence.

### Phase 1: Pandemic shock: Uncertainty of the unknown

The first phase experienced by the healthcare workers was that of pandemic shock, and the theme of the uncertainty of the unknown emerged in this phase. FHCWs described their early pandemic experiences with terms referring to the shock of a totally unknown situation and a lack of understanding of the disease. Six concepts under uncertainty were: “Worry and fear,” “Lack of Knowledge,” “Lack of Information,” “Lack of Adequate Supplies,” “Stigma & Discrimination,” and “Feeling Vulnerable.”

Everyone expressed a fear of what could happen next, the need to find out from others and from the emerging evidence of what the disease progression looked like, and what risks were attached. Many of the FHCWs spoke of how they thought they were becoming COVID-19 positive, with the fear of uncertainty. For example, one participant shared her earliest feelings about the uncertainty:

“Somehow it seems that we are waiting for our turns to become COVID-19 positive and then get cured by the grace of the Almighty.” (P5)

Working in a humanitarian setting during the pandemic was characterized by a lack of knowledge, information, and uncertainty around supplies on COVID-19, which caused a great deal of distress. The lack of PPE was concerning for participants, especially when they were unsure whether they would have enough masks or gloves. A few participants mentioned the hot humid weather in the camps, which was even more higher inside the health centers. This also made the use of PPE even worse. The FHCWs experienced vulnerability to many negative physical, social, and emotional responses during the earlier stages. Most of them were young and stayed alone in the camps without any of their family members nearby.

### Phase 2: Pandemic awareness: Beginning to notice

In phase 2 of the study, health workers underwent a process of pandemic awareness, characterized by the theme of “Beginning to notice.” Within this phase, three key concepts were identified: “Being Self-Aware,” “Mastering Self-Preparedness,” and “Engaging in Self-Protection with responsibility.” FHCWs started to notice what was going on around them and consciously chose to learn to prepare themselves to confront the pandemic. The COVID-19 pandemic had a physical and emotional toll on the humanitarian workers, and community members saw them as high risk, adding to the negativity in their already difficult lifestyles. Participants emphasized the importance of understanding personal and professional perspectives to grasp their inner feelings and intentions. FHCWs believed that successful confrontation of COVID-19 requires engaging in self-protection and responsible behavior. The pandemic awareness process led to the maturation of self-awareness, as health workers learned from their own experiences and versatile information through workplaces, newspapers, and media. This led to being more inventive and intentional in the structuring of daily lives. Some generated their own set of personal values, beliefs, and behavior, while others found comfort in pursuing religious involvement.

Participants highlighted that community people saw them as high risk, and that added some more negativity in their already difficult lifestyles. For example, one physician participant added, “It's like a crisis within a crisis.” (P1)

A community health worker shared this: “As a regular routine, I commence my workday by arriving at the office at precisely 8:30 in the morning. Subsequently, I proceed to engage in my daily tasks, which involve fieldwork for the entirety of the day. During these field visits, I conduct home visits for expectant mothers, providing them with necessary health care services. Specifically, upon visiting 20 households, we offer counseling and guidance to these families and refer them to our health post. Here, a team of healthcare professionals consisting of a physician and a nurse provide essential medical services and administer prescribed medication.

I cannot emphasize enough how crucial it is to use caution and prioritize safety in all facets of life as someone who is currently experiencing the ongoing global health catastrophe. Because the virus is so common and can spread by simple motions like sneezing and coughing, it is crucial to take precautions to protect oneself. I have been avoiding busy areas, avoiding face-to-face interactions with people, and avoiding being in close contact to others as advised by health professionals and authorities. Because it is unknown how infected the persons involved are, interpersonal communication that used to take place in close quarters now poses a serious risk. I have been keeping a safe distance in order to protect my own safety and reduce the possibility of negative repercussions.

In my own line of work, our morning session typically only lasts 20-30 minutes if we're simply signing in and moving on. Once we finish that session, we head out to the field to carry out our work. There simply isn't any time to hang out given our busy schedules and the current situation. Instead, we remain focused on our work, completing the necessary tasks and duties in a timely and efficient manner. After working in the field, we report back to our team and continue to prioritize safety measures in order to help mitigate the risk of contracting or spreading the virus.

I put away my work clothes in a specific spot when I get home from the office and change into new clothes. I then wash my previously worn garments as necessary. It can be difficult to tell who may be positive or negative for COVID-19 because it spreads by respiratory droplets, so it's important to take precautions and take care of your personal cleanliness. Using protective gear, regularly washing one's hands, and avoiding close contact with others can all assist stop the spread of the illness and advance personal safety.”

There was not one specific event or activity that changed perceptions, instead it was a combination of the ongoing nature of the pandemic and the witnessing of others struggling with the endlessness of it that brought about change. When the pandemic began global and national messaging was focused on the emergency nature of the pandemic. The realization that the emergency was different in its longevity and that it was all encompassing - infiltrating into every aspect of life helped FHCWs to take stock and notice.

### Phase 3. Pandemic learning: Realizing seriousness

The third phase is best depicted as “Realizing Seriousness,” which had four subthemes: “Importance of Training,” “Self-directed Learning,” “Organizational Support,” and “Work-life Balance.” In this phase, most participants were still learning about COVID-19 and did not feel confident while making self- or patient-management decisions.

FHCWs spoke of the importance of educating the entire group of FHCWs, creating public awareness about what might happen during a disaster because of the unavailability of resources, or the availability of extremely limited resources because of devastation during an emergency. Thus, more efforts need to be pot toward customizing and designing the training and education of FHCWs, which can be shown to lead to effective implementation of self-preparation practices for the pandemic.

One nurse said, “I know less about the disease and situation management.” (P 24)

One physician described his experience, “I am aware that stress, in moderation, can be beneficial in my work. Yet I'm also unsure of my safety and whether I'm following protocol correctly. One area where I feel particularly anxious is donning and doffing personal protective equipment (PPE). Even though I've been trained on the proper techniques for donning and doffing PPE, I'm still concerned that I might make a mistake and unintentionally expose people to the virus. The consequences of such a mistake could be dire, not just for myself but also for my colleagues, family and patients. The pressure of ensuring that I follow protocol properly and maintain a high level of vigilance can be overwhelming at times.” (P6)

They emphasized that learning enhanced their self-esteem and confidence. Learning is also a social process of constructing shared meanings and beliefs, which is a result of social interactions that are necessary for individuals to interpret and give meanings to their experiences. In the reality of the Rohingya refugee camp situation, FHCWs needed to pick and adopt and get habituated with all kinds of self-protective measures, which they did through their own learning. Taking responsibility for their own education was one part of the process of engaging in self-protection with responsibility.

Many participants spoke of the need to protection should not only be applicable for the workplace and the need to change peoples' perspectives. There was a strong message among all FHCWs that they must wear their PPE all the time to break the cycle of getting in contact with an infected patient, but it was a difficult task to carry out. There were challenges in receiving PPEs, although this was resolved as time went on.

The balance between professional responsibilities and personal responsibilities was another area difficult to maintain. One physician shared his harrowing experience trying to get his pregnant wife medical attention during a lockdown in early pandemic situation:

“That whole time was like hell for me. That time... It seemed like... those few days, and then the two weeks after that were completely bad for me. That day was particularly the worst.” (P7)

He assumed his wife had viral hepatitis because of her yellow eyes and high fever, therefore he had to take her to Cox's Bazar while he was still living in Ukhia. He was able to get a test done that showed his wife had a bilirubin level of 9.2, which indicated viral hepatitis, despite having trouble accessing transportation and medical care. The COVID scenario made matters worse, so the couple was forced to spend the night at a colleague's home even though the owner of the home claimed they couldn't accommodate guests because of the pandemic. They had to obtain an ultrasound the following morning and go back to Ukhia.

“The worst day was that one in particular. As it happened, we realized that for those two weeks, we were unable to perform even basic tasks. In addition to working and cleaning the house, I am also shopping and cooking. Due to this COVID, no maid will be arriving. She is already pregnant with viral hepatitis, and since I will be in close contact with the patients, if I am working as a carrier or whether it is already spreading from me. Also, if I act as a carrier, will I infect her if I do so?” (P7)

### Phase 4: Pandemic resilience: Practicing strength

The fourth phase was that of pandemic resilience, captured through the themes of “Advancing Self-Competencies,” “Courage and Willpower,” “Self-Confidence,” “Coping strategies and resources,” and “Opportunities to act as a FHCW.”

Resilience incorporated the idea that participants recognized the suffering and were actively concerned about the continuation of their activities forward. The FHCWs were no longer the ones with a fear of uncertainty. Because of self-competencies and self-confidence, a bond was established which helped to look toward opportunities within the pandemic and struggles. One participant described the situation as:

“If everyone walks behind, it will not be possible to face the pandemic.”(P12)

The participants discussed different coping strategies and resources to engage in resilience: “breathing exercise, communication with family and friends, debriefing at workplace, family support, listening to music, meditation, physical exercise, and work/life balance.” In addition, the FHCW's belief was about what was important in life changed, with many saying that they no longer worried about “getting COVID positive.” These beliefs appeared to be fundamentally influencing the professional self-preparation construction and resilience in FHCWs.

### Phase 5: Pandemic resurgence: Advocating for self and others

In the final phase, nearly all participants took the lead in their self-management and developed an understanding of the multifaceted impact of COVID-19. The participants shared numerous strategies when advocating for themselves and others. A set of comments which resonated among many healthcare workers were “commitment to a life of purpose,” “prayers,” “adapting to change,” and “cultivating skill & expertise” for self-preparedness. Participant 1 discussed the theme “Advocating for self and others” as an important rationale to engage in self-preparedness, which also meant being sensitive at all times to the risks of the pandemic.

“People feel helpless of being infected with COVID-19, so to serve them as well as protecting ourselves, being fully aware about self-preparedness is our commitment.” (P1)

The participants identified that they needed to continue training and counseling for prevention and for staying safe from COVID-19. The participants reported that they felt vulnerable and powerful, stressed, and had personal satisfaction at the same time. The ultimate success was the subtheme “Personal insecurities turn into professional identities.”

One nurse described in his words: “We've been battling this pandemic for more than a year now. And even though we've made certain adjustments, maintaining this way of life is still challenging. We need to take extra care every day to protect our loved ones and ourselves. It exhausts you physically and mentally. But, we must continue since it is our duty and responsibility as healthcare professionals. No matter what, we have to keep both our patients and ourselves safe.” (P17)

Another participant, a physician, who had experienced a challenging situation explained: “I learned a lot from that incident, and I am grateful for the help that I received from many people during that time. When everything started shutting down due to the pandemic, I realized the importance of having a support system in place. My colleague was one of the people who helped me out a lot during that time, and I am thankful for their assistance.

Another lesson that I learned was the importance of having a network of reliable contacts, especially in the village where I live. In case of any emergency, having someone to call for help or support can be invaluable. I now make sure to keep my phone number and a few vehicle contacts with me at all times.” (P7)

Table 2 shows some representative quotes from the study.

**Table 2:** Representative quotes.

**Phase 1: Pandemic shock: Uncertainty of the unknown**	
(-) Uncertainty	“Somehow it seems that we are waiting for our turns to become COVID-19 positive and then get cured by the grace of the Almighty” (P5)
(-) Anxiety	“Actually we are working on the frontline, we are all living with this anxiety” (P16)
(-) Fear	“Now what will happen to me? Am I going to die? Moreover, I'd need to be in isolation area, there would be no one there. That means you have to survive there alone.” (P19)
(-) Separation from family	“For the last 4 months, I could not go to Dhaka to see my parents.” (P3)
(-) Lack of adequate supply	“For the first 15 days, we didn't have any PPE” (P15)
(-) Feeling vulnerable	“My wife (also a doctor) was pregnant. It was an early pregnancy, only 4 weeks. Suddenly she had started fever, vomiting, and jaundice. I thought it was viral hepatitis and we desperately needed to check. There was no testing facility in camps. It was lockdown everywhere. I had to take her in Cox's Bazar that's 35 km away for testing with an auto rickshaw.” (P6)
(-) Stigmatization and discrimination	“Needed overnight staying at a colleague's house in CXB, his landlord forbade him to allow us to stay” (P6) “House owners have stopped to let their houses to the clinicians, fearing they are sources of COVID-19” (P14)
**Phase 2: Pandemic awareness: Beginning to notice**	
(-) Context realization	“It's like a crisis within a crisis” (P9)
(-) Lack of knowledge	“I know less about the disease and situation management.” (P 14)
(-) Asymptomatic cases	“As a frontline worker, since I've been in touch with a lot of patients, even after having protection, I feel worried that I may get infected anytime.” (P11)
(-) Limited testing facilities	“Since testing facilities are still very limited in our country and we are not able to bring everyone under testing” (P7)
(-) Lack of awareness	“Everyone wears a mask but doesn't know how to wear it and how to remove it. Many people wear masks that are so dirty. People are wearing gloves but tearing parts of fingers, since it is difficult to press the mobile. We need to be more cautious about hygiene and correct use.” (P22)
(-) Physical weakness	“I'm having a lot of physical problems. I feel weaker than before because now I'm wearing this heavy PPE during duties in a hot climate in Rohingya refugee camps. I'm having a continuous headache. I'm dehydrated. I'm having problems with my BP; my BP is getting low due to dehydration.” (P4)
**(-)** Stigma at community	“When the camp dwellers saw us wearing PPE, they got scared and avoided us. They used to hide their symptoms” (P29)
(+) Organizational support	“The organization where I work has some good practice and organizes monthly or fortnightly sessions where COVID-19 related issues are explained” (P1)
(**-**) Staff shortage	“We are not just working with COVID now, general patients, the elderly, and children are also getting treatments. In that case, it would have been better if the number of health workers could be increased in this situation.” (P8)
**Phase 3. Pandemic learning: Realizing seriousness**	
(+) Create awareness	“We need to raise more and more public awareness. Repeated counseling, more publicity in the media about what to do and how to do it, are urgently needed.” (P13)
(**-**) Lack of training	“Although I'm working in this Coronavirus pandemic situation I haven't received any training yet.” (P11)
(**-**) Everyday challenges	“In fact, the extremely hot weather in Rohingya camps is very difficult for a health worker to wear this. One who has worn it will understand how difficult it is to keep wearing it. And the most important thing is that you can do easily the donning part, but the real difficult thing is at doffing. After work, as it is very hot here, when we go out to work, we are in a panic mood. So, then we try to remove these from our body quickly. As a result, we are more likely to be infected with this doffing.” (P2)
(**-**) Frustrating reality	“We are wandering around, not believing anything. I'm not wearing a mask; I'm sneezing and coughing, not bothering about cough etiquette. People rushed home without accepting anything during Eid holidays. People are attending wedding parties.” (P17)
(**-**) Heavy workload	I work with 12,000 people (among the total population of 44,000) in my camp. I have to go to camp daily and search for symptoms, that are divided into yellow, red & green, in them. I have to cross mark in the tally sheet. I submitted 28th report last week” (P28)
(-) New realities	“When we go to the camp through the vehicle, we sit at a distance, when we reach the workplace there is chlorine solution mixed. We wash our hands, we wash our legs, we have to go through thermal screening.” (P26)
(-) Isolation Centre	“We visit patients at the isolation centre after wearing PPE. At the end we do “doffing.” We have an observer who tells us the steps to follow, at the end we place our gumboots into chlorine wash.” (P27)
(-) Burnout	“And actually, returning home from outside is now a hazard. Previously, returning home was a joy and relief. And now when I get back home, the first thing is to think about my hygiene and my sanitation. Wash my hand, and shower, sanitize all my stuffs that I'd taken outside.” (P10)
**Phase 4: Pandemic resilience: Practicing strength**	
(+) Coping strategies	“I do breathing exercise sometimes. When I am anxious, I take 3 deep breaths and release slowly. Apart from this, I try to feel every part of my body closing my eyes, which I've learnt from a psychosocial training. I try to do this when I am very stressed, which gives me a bit of relief.” (P6)
(+) Self-directed learning	“I completed training from my personal initiative such as I've completed online courses offered by the World Health Organization. I try to keep myself up to date reading journal papers.” (P11)
(+) Family support	“I got a lot of support from my family. I talk to my parents everyday over telephone. That gives me mental comfort.” (P8)
(+) Monitoring awareness	“As a health worker to prevent coronavirus infection, my opinion is that now the only way to prevent it is for everyone to be aware of themselves to protect themselves. As a health worker, I need to make others aware of this.” (P15)
(+) Communication in camps	“In the beginning, the camps were very crowded. We told them not to visit another house. If someone was getting married then not to join the party. Don't gather and gossip in the store. Because if you keep yourself safe, your family will be safe.” (P21)
(+) Self-confidence	“Now I am fully prepared, now we have proper supply of everything. And we're keeping a distance of three meters, everyone is wearing a mask, moreover no one has any plans to visit other places for now.” (P13)
(+) Faith and self-responsibility	“When I go to work, my actual protection is PPE, which covered my head to toe, which we call coverall, N-95 mask, goggles and face shield. Outside the work area I usually use masks and goggles that I've bought on my own. So far, I'm healthy. By the grace of the Almighty, I'd say those are protecting me.” (P12)
(+) New normal	“After returning from outside I go straight to the bathroom and dip clothes in soapy water. After cleaning these, I take a bath and then come in contact with others.” (P25)
(+) Courage and willpower	“Since I am a nurse, we have to fight as front liners. We should not be afraid. We will have to continue to face this situation today and tomorrow. If everyone walks behind, it will not be possible to face the problem.” (P20)
(+) No absenteeism	“Since we are working as a front liner we are getting infected. I have not been able to go to work for last 14 days as my COVID test was positive. Those who came in contact with me were also kept in quarantine. This was not intentional. Everyone tries to work with self-protection responsibility as much as possible. Besides actual reasons, no one remains absent in workplaces.” (P10)
(+) Resilience	“Many of my colleagues often get upset while working. I try to counsel them. I describe the reality of the situation with what we are working and also that we have to continue the work. So we should take care of our physical and mental conditions. Because if we are not healthy ourselves then how would we provide proper services to the patients? This is why we have to stay healthy.” (P11)
(+) Opportunities to act as a FHCW	“I'm not worried when I am on duty. Actually, I say it from the context; the highest numbers of health care workers in Bangladesh getting infected are who work at Non-COVID units. The reason behind this is when we work in COVID unit we maintain 100% protection. But those who are working in Non-COVID units, they do not get proper PPE or ignore the use of PPE. There are so many who comes to Non-COVID unit for the treatment who hide their symptoms so that they are at much more vulnerable situations.” (P16)
**Phase 5: Pandemic resurgence: Advocating for self and others**	
(+) Commitment to a life of purpose	“We have taken the Hippocratic Oath right, as our patients would be our first priority” (P5)
(+) Prayers	“The biggest learning from this corona time is we can control ourselves. The situation in the country and abroad are going deadly. In this case, if I keep myself panicked, I cannot provide my service properly. As a physician I get many phone calls from people with COVID-19 symptoms, all of which I try to attend. I feel like, thanks to the Almighty, that HE has given me the capability to help others. I pray to maintain this and gear myself up psychologically.” (P1)
(+) Cultivating skill and expertise	“Now we know how to prevent it, if we are on pause, the prevention will be also at pause. So we need to arrange more training and let people know and help them to understand through counseling and inform everyone how to stay safe from it, there will be a change of situation into ‘less fear and more courage' will happen then.” (P9)
(+) Adapting to change	“As a health professional and human being, in both cases I see myself in a vulnerable group. Because I am going to the forefront everyday but at the same time I see myself again as a powerful source that I have got a chance to fight this pandemic, when the world needs more people to come forward and help each other and at least I have some medical knowledge which would make a difference here.” (P5)
(+) Personal insecurities turns into professional identities	“And in the time of crisis for a life-a human life, I can make a difference that also makes me feel content and satisfied. So even though I'm vulnerable, feeling stressed and I'm happy being in a position where I can give my support and care.” (P7)

**Figure 3:**
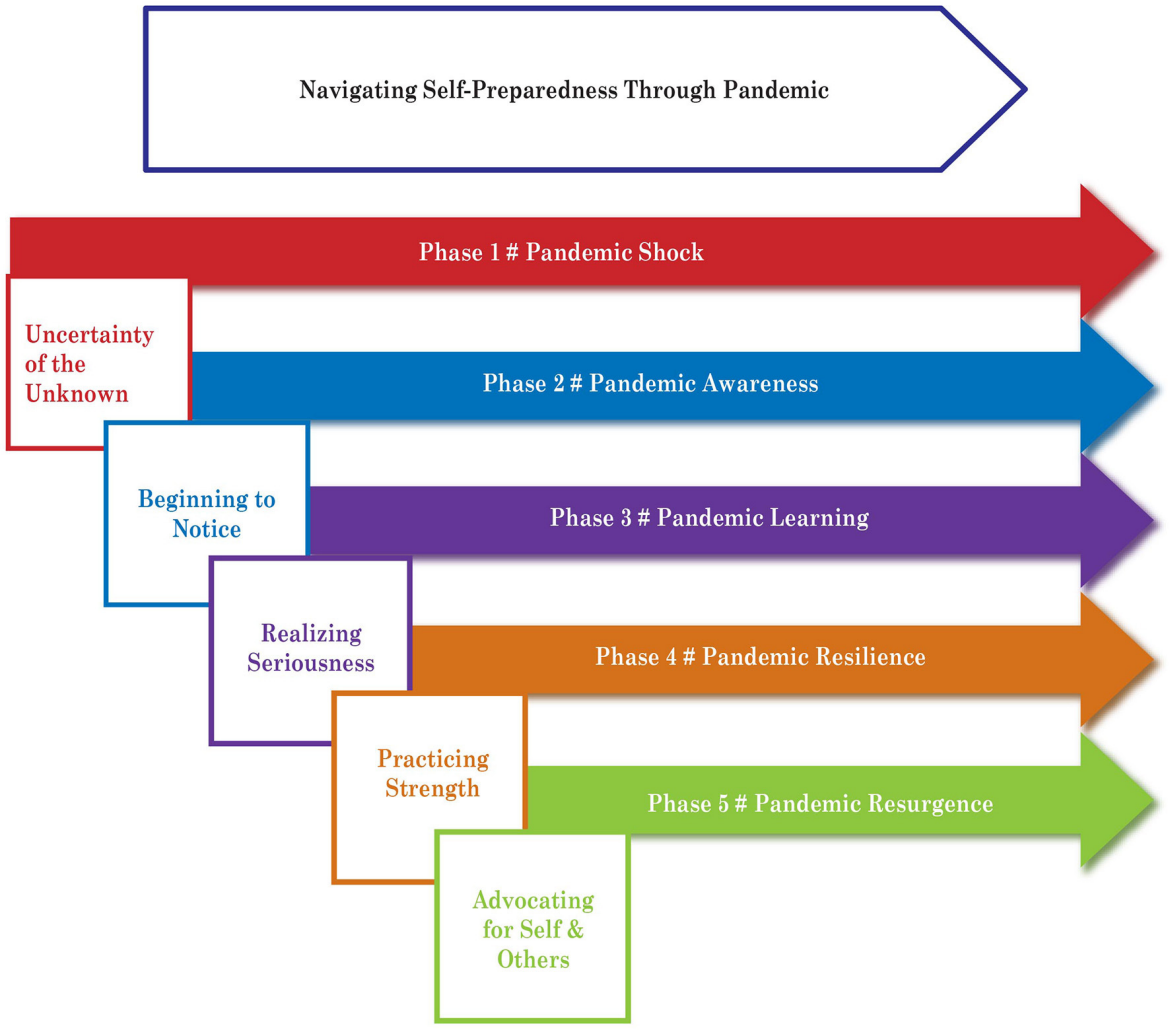
Constructed theory.

From these phases, the emergent-grounded theory of “Navigating Self-Preparedness Through Pandemic” was developed (Figure 3). The theoretical framework established a realistic, socially, and culturally sensitive COVID-19 strategy to protect and support FHCWs.

## Discussion

Self-preparedness of FHCWs in the humanitarian context is an innovative and important area to be explored further. This research has used a unique lens to view an inter-professional team of FHCWs' understanding, knowledge, and perceived behavior of self-preparedness in a humanitarian setting in a lower middle-income country during the pandemic. The term “self-preparedness” denotes a set of competencies including managing self and others, nurse coping with ambiguity and uncertainty, communication, critical reasoning, continuing own education, identifying, analyzing and solving problems, and practicing empathy. This study adds to the existing body of knowledge by expanding upon the experiences of the daily life span of FHCWs not only within the workplace. Furthermore, the study explored the mental, social, and spiritual aspects of FHCWs' experiences and how their self-care perception changed and strengthened over time in response to the COVID-19 pandemic, providing valuable evidence of strategies to measure and manage future outbreaks with the support of healthcare workers.

The knowledge that human-to-human transmission is likely to be exacerbated and accentuated because of the heavily congested nature of the Rohingya refugee context, where over 1 million people were living in makeshift tents, added to an important dimension to undertaking this study. The Rohingya refugee camps were in a uniquely vulnerable position, and frontline healthcare professionals have been working under enormous pressure, often with limited resources to respond to patient needs. The research exercise allowed the participants to share with the world their immediate experience of caring.

While there is emerging literature on the importance of personal protective equipment to help healthcare workers in this pandemic, there is little information on the way that healthcare workers understand self-preparedness, the strategies they use to self-protect, and the complexity of self and professional care.

The primary objective of this study was to develop a theoretical model explaining the phases of the processes of engagement in self-preparedness for FHCWs working in humanitarian camps. The theory “Navigating Self-preparedness Through the Pandemic” provides a way of understanding how the study participants engaged in the process of self-preparedness for self and patient care in the humanitarian context. The theory represents the transition of participants moving toward the process of self-preparedness to achieve the desired outcome.

As Figure 1 illustrates, this study sought to clarify the shifts as healthcare workers went through five consecutive phases. Once the process of uncertainty began, awareness, learning, resilience, and resurgence occurred progressively. After the first-phase encounters of the pandemic by the FHCWs, it was crucial to continue to manage self and manage service provision, mainly getting back the courage and willpower to embrace the vulnerability of life. Every human being is unique and they have their own needs, preferences, and choices in the process of preparing for self-protection. We approached the research with these values in mind. The combination of symbolic interactionism, social constructivism, and the grounded theory methodology was crucial in helping the researchers to uncover, understand, and explain such modifications.

Phase 1 describes the process of engaging with the uncertainty of the pandemic while the burdens of anxiety, fear, burnout, and depression were becoming more predominant as workers were trying to cope with unyielding odds. It helps to understand the stresses and vulnerabilities of the participants. The experiences of the pandemic were new to all the participants. Of all the participants in our study, only a few had a little textbook knowledge about the pandemic. It also highlights the anxieties and the risks that healthcare workers felt. This was evidenced among the citations of the participants (Table 2).

Phase 2 outlines the gradual process of how the initial shock of uncertainty led to an awareness build-up that started with knowing one's self-role and engaging in self-protection with responsibility. The FHCWs' acceptance of dreadful circumstances appeared to be a prerequisite to reflecting on their awareness of the situation. Acceptance of anxieties and worries involved recognition and awareness that they were living with the possibility of contracting COVID-19 at any time. It was the opposite of denial or feeling that it was not happening to them. The participants' statements reflected a variety of feelings associated with the seriousness of COVID-19. The FHCWs realized the need for their presence in the frontline, which was not only being there but was immensely important.

In phase 3, the participants slowly started realizing the seriousness of the situation. They associated learning with a wish to do something for the patients. Many infected patients had none or very subtle symptoms and some exhibited atypical symptoms. They were aware that such patients greatly endangered the health of the staff even though clinical areas caring for patients with and without COVID-19 were separated from each other with isolation centers in place in refugee camps. The FHCWs became aware of the importance of acting toward protecting and improving their own health as well as becoming supportive of healthcare activities. A majority of the FHCWs mentioned having a dedicated, supportive, and caring working environment through organizational support; adequate PPE support; reasonable working hours; and regular training helped them to face the pandemic. They also opined to increase the number of workers as a key factor similar to other studies (14).

In phase 4, participants began to experience self-confidence and a sense of life changes after the recognition of opportunities as a FHCW. In phase 4, they developed trust in the team and learned about self-management practices from training. For many, the pandemic resulted in increased self-confidence that manifested as a greater perceived ability to achieve their needs and goals. During the course of their activities during the pandemic, most FHCWs reflected upon the sufferings they experienced. Consequently, they were able to reflect upon and consider their pandemic suffering as something different from everyday living, thereby experiencing a different and new perspective on their lives. In particular, the exceptional sufferings of “stigmatization and discrimination” could be cited here (Table 2). They used physical exercise, yoga and meditation, team support, religious activities, and learning about COVID-19 as their personal coping strategies (15).

Nearly all participants in phase 5 were able to make spiritual connections through “prayers” with the appreciation of the circumstances and were “cultivating skills and expertise” to take immediate action if it became worse and adapted themselves to change. Ultimately, the skills and expertise acquired during the learning process and the experience of applying self-preparedness improved their commitment to a life of purpose. The FHCWs were concerned for themselves, for others with compassion, and for the hope that still was present. Their sense of duty as compassionate healthcare workers and as people who cared emerged strongly as did their fear and anxiety in the face of the unknown nature of the pandemic, especially given the minimal protective support that they had in the early period of the pandemic.

During a pandemic, difficult choices have to be made about how to secure the best health outcomes not only for patients and communities but also for FHCWs. The findings from this study highlight the need for the delivery of education and training programs to build up self-preparedness. There is also a need to facilitate FHCWs transition throughout the process of engagement in self-preparedness. Engaging individuals in self-preparedness indicates supporting them as they navigate the daily management of self and others. It involves preparing and supporting FHCWs to participate in self-preparedness as early as possible and as much as they desire and to their ability. Further research and development are critical to have an informed and evidence-based response.

### Limitations of the study

This study has several limitations. We used the “convenient sampling” method, which is a potential limitation of the study. However, we recruited three different categories (doctors, nurses, and CHWs) to represent the “frontline healthcare workers” and undertook in-depth interviews. The interviewees took part with total autonomy.

We recognized the challenges of recall bias although information collection took place quickly, and we surmised that the possibility of recall bias was small. The study cannot be generalized as the research was qualitative, which focused on a small number of subjects located in a particular setting. However, our aim was to generate a theory to generalize rather than generalize the findings to a wide context or population.

Many qualitative studies have been criticized as “too subjective” without separation of interviewer and interviewee considerations and danger of bias from the same interviewer given how researcher-dependent qualitative studies are. To minimize the potential limitations, our study used five researchers from different backgrounds with different experiences and perspectives, theoretical sampling, and coding procedure to direct and be grounded in participants' perspectives.

### Clinical implications

These data highlight the urgent need to implement policies to better protect FHCWs and to better understand the drivers that enable healthcare workers to keep going and to self-prepare themselves physically and mentally in a comprehensive way. FHCWS had to constantly modify their behavior and actions within their social milieu and to reconstruct their reality through daily management of their condition. Therefore, their voices need to be listened to explore the factors that have influenced their ability to engage in activities in humanitarian settings.

The way FHCWs negotiated, managed, and adapted to the significant stresses within their lives and environment from phases 1 to 3 facilitated resilience to bounce back in them in phase 4 (16, 17).

This study suggests that self-preparedness was not simply a matter of individual FHCWs changing their behavior. The willingness to participate and the information flow about self-preparedness and knowledge was spontaneous while living during the peak time of the pandemic. Having a self-preparedness approach helped individual FHCWs to feel that their views and concerns are making a difference in the overall humanitarian health context, despite the existing barriers of the pandemic such as lockdown and social distancing and ultimately contributing to a collective strength as a public health measure.

## Conclusion

Globally, it is deeply concerning that our knowledge about the physical, mental, social, and spiritual wellbeing of those upon whom the world depended to manage the pandemic is limited. Unfortunately, our knowledge is even more limited about the needs of those working in situations of fragility, such as humanitarian settings during the pandemic. This study has shown that FHCWs have much to gain by reorienting their anxieties and fears into concrete thoughts of learning and resilient actions of self-preparedness toward systematic prevention, protection, and improvement of humanitarian health settings in a pandemic. As the pandemic progressed, there were profound changes in the way that FHCWs thought about their health and role. We believe that the distinction FHCWs made between “pandemic shock” and “pandemic resurgence” warrants further investigation, particularly in a conflict-affected humanitarian situation.

## Data availability statement

The raw data supporting the conclusions of this article will be made available by the authors, without undue reservation.

## Ethics statement

The studies involving human participants were reviewed and approved by IRB of University of Edinburgh. The patients/participants provided their written informed consent to participate in this study.

## Author contributions

FK: conceptualization, funding acquisition, project administration, methodology, data curation, formal analysis, writing original, and final drafts. TA: methodology, supervision, project administration, data curation, read the first draft and provided feedback, and read final manuscript. SB: methodology, data curation, software management, read the first draft, and read final manuscript. HK: supervision, data curation, read the first draft, and read final manuscript. LG: methodology, development of themes, contributed to writing, and edited and reviewed the final manuscript. All authors contributed to the article and approved the submitted version.
